# Effect of high-protein vs. high-fat snacks before lunch on glycemic variability in prediabetes: A study protocol for a randomized controlled trial

**DOI:** 10.3389/fnut.2022.925870

**Published:** 2022-07-19

**Authors:** Yupeng Liu, Huinan Jiang, Binye Ruan, Yi Liu, Siyu Le, Xiaoyi Fu, Shuran Wang

**Affiliations:** ^1^Department of Epidemiology and Biostatistics, School of Public Health and Management, Wenzhou Medical University, Wenzhou, China; ^2^Department of Nutrition and Food Hygiene, School of Public Health and Management, Wenzhou Medical University, Wenzhou, China

**Keywords:** glycemic variability, pre-meal, snack, high-protein, high-fat, randomized controlled trial

## Abstract

**Background:**

China has the largest number of patients with Type 2 Diabetes Mellitus (T2DM), and it tends to increasingly grow in the future, putting an enormous burden on disease control and prevention in China. While glycemic variability (GV) came to be an important indicator of blood glucose control in diabetic patients, studies suggested that premeal snacks may help blood glucose control, but there are still some problems to be researched. Therefore, we designed this trial to evaluate which kind of premeal snacks would lead to better effects on GV under two diet patterns in pre-diabetes subjects and to evaluate assessments of acceptability and compliance, behavior, and metabolism changes in individuals will be described.

**Methods and analysis:**

The study is a single-center, open-label, multiparallel group, randomized controlled trial. A total of 32 male and female volunteers will be randomized into 4 groups in a single allocated ratio of soy milk (powder) snack, milk (powder) snack, almonds snack, and placebo control with 250 ml of water taken 30 min before lunch, respectively. The study consists of two intervention periods over 11 days. The first intervention period under habitual diet conditions from D3 to D6 (4 days), during which all subjects are asked to maintain their habitual eating and daily activities similar to the run-in period. The second intervention consists of prelunch snacks with standard meals. We will examine both the effect of GV and various metabolic and behavioral outcomes potentially associated with the interventions. At the end of this study, we will assess the acceptability and maintainability of the intervention through interviews.

**Clinical trial registration:**

Chinese Clinical Trial Registry, identifier ChiCTR2200058935.

## Introduction

The 2018 China National Survey reported that the prevalence of diabetes mellitus among adults aged 18 years and older in China was 12.4%, and estimated that the number of adults with diabetes has reached 114 million, with China having the largest number of people with diabetics worldwide. Furthermore, 38.1% of people are prediabetic, indicating that a large number of people are at high risk of the future development of type 2 diabetes (1). The glycemic profile of diabetes is characterized by chronic persistent hyperglycemia, hypoglycemia, and acute glycemic variabilit (GV). The hemoglobin A1c (HbA1c) has been commonly accepted as the gold standard for chronic persistent hyperglycemia, and reflects the average blood glucose status over the past 2–3 months (2). However, even in patients with similar HbA1c, within-day GV may be different (3). Observational studies with human populations and experimental studies with animals have found that GV is positively associated with diabetic retinopathy, which is an independent risk factor for cardiovascular autonomic neuropathy in patients with new-onset type 2 diabetes, increases all-cause mortality in critically ill patients, and has negative effects independent of chronic persistent hyperglycemia by activating oxidative stress, impairing endothelial cell function, and exacerbating inflammatory responses (4–8). The American Diabetes Association (ADA) 2020 Standards of Medical Care in Diabetes also include a Time in Range (TIR) >70% as one of the key indicators for glycemic management due to the strong correlation between temporal TIR and HbA1c in the range of GV indicators (9). Therefore, improving GV is important to delay the development of diabetes and its complications and to improve the prognosis and quality of patient survival.

GV is defined as both upward (postprandial) and downward (interprandial) acute variability of glucose around a mean value (10). Researchers have attempted to lower peaks and raise nadirs through various dietary interventions, such as changes in meal timing, meal order, carbohydrate quality, quantity, or distribution throughout the day; protein intake, dietary fiber intake and macronutrient ratios (11–14). Among these interventions, premeal snacking is the simplest intervention that has attracted attention as it does not require major changes to overall eating habits. Studies have attributed the benefits of premeal snacks to the second meal effect, which is defined as the effect of a prior meal on reducing postprandial glycemic excursions (15). A growing body of recent studies has used the “second meal effect” theory to intervene in blood glucose variability (16, 17). Many previous studies have focused on single nutrients as premeal interventions. It has been suggested that the use of an intervention with a small dose of whey protein before a meal is more effective than interventions that use soy protein for improving appetite, energy intake, and body composition (18). However, this finding is somewhat inconsistent with the results of an 11-year longitudinal study of the effects of different food sources of protein on metabolic syndrome, which concluded that a diet high in plant protein and low in animal protein helps prevent metabolic syndrome in healthy people (19). The results of a meta-analysis showed that fasting blood glucose, HbA1c, and fasting insulin levels were significantly reduced in patients with type 2 diabetes when the daily intake of soy products accounted for more than 35% of the total protein intake (20). In addition, nut consumption may reduce the risk of T2DM and cardiovascular disease (CVD) by improving glucolipid metabolism, maintaining body weight, and improving endothelial function (21). Several studies have found a significant effect of a nut-rich diet on glycemic control in patients with obesity or adults with type 2 diabetes (22–24). The long-term intake of almonds, which are widely accepted as snack, has been suggested to improve glycemic control and lipid profiles in patients with T2DM (25). Recent advances in appetite research also demonstrated the effectiveness of raw almond intake in healthy females (26). However, there is still a lack of randomized controlled trials addressing the effects of nuts as premeal snacks on GV.

In studies of the second meal effect, the meal pattern is also very important to the results. Most of the currently proposed premeal intervention studies are conducted under well-controlled laboratory conditions with uniformly balanced diets or use glucose tolerance tests to simulate eating behavior. Although the effects were obvious, it is not clear that similar benefits can be achieved under real daily diet conditions. Moreover, single nutrients or intervention foods in experimentally specific ratios are not readily available in daily life and are difficult to adapt to the different dietary habits of people in China and other regions, which may lead to poor adherence. Therefore, we will select easily accessible natural foods, i.e., soy powder, milk powder, and almonds, to represent high plant protein, high animal protein, and high fat premeal snacks intervention, respectively, and evaluated their effects on GV under two different dietary patterns: habitual diets and balanced standard meals. In this trial, we will fully consider dietary habits, offer common foods as premeal snacks without energy restrictions, and detect the effects on GV and energy intake. Our trial is expected to have high applicability, operability, and acceptability, which can provide evidence for further application in T2DM populations.

The primary aim of this study is to evaluate whether premeal snacks of natural foods improve GV and which kind of premeal snacks lead to better effects in prediabetic subjects. We hypothesize that plant-based high-protein snacks will lead to a non-inferiority effect of animal-based high-protein snacks on GV. The secondary objective is to evaluate whether these interventions have a positive effect on insulin, HOMA-IR (homeostasis model assessment for insulin resistance), HOMA-β (homeostasis model assessment for β cell function), C-peptide, body weight, waist circumference, abdominal circumference, subjective appetite, hunger, and satiety. Furthermore, we will assess the acceptability and maintainability of these interventions.

## Methods and analysis

### Study design

**Figure 1:**
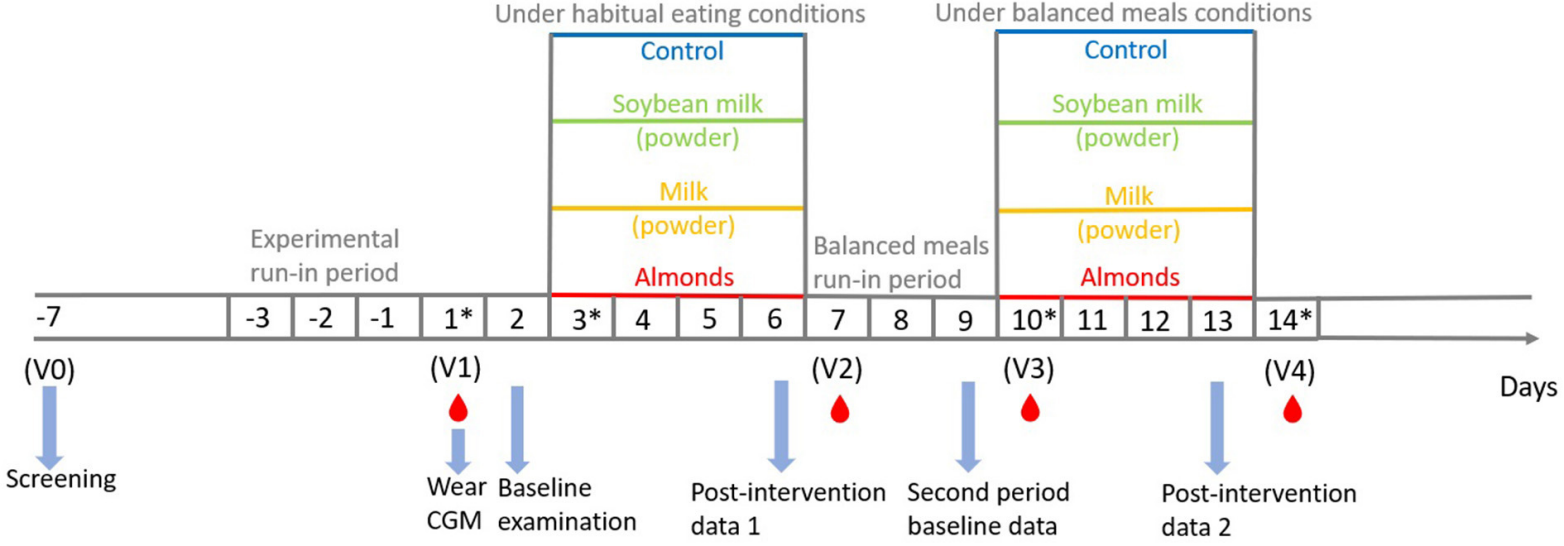
Study design. *Data from CGM are excluded from the final analysis. CGM, continuous glucose monitoring.

This study is a single-center, open-label, multiparallel group, randomized controlled trial. The study consists of two intervention periods: [1] under habitual diet conditions and [2] under balanced standard meal conditions. After a 3-day experiment run-in period, a total of 32 subjects (16 males and 16 females) will be randomized into one of 4 arms in 1:1:1:1 ratio (Figure 1). The 4 arms will include soy powder and milk powder which will be completely dissolved in 250 ml water, almond snacks with 250 ml of water, and a control arm (250 ml of water). Test days will be scheduled before and after the two intervention periods to assess the potential effects of premeal snacks under the habitual diet and standard balanced diet patterns, respectively. During the entire period of this experiment, subjects will be asked to complete a food recording diary (online) including the type, amount, and time of all meals, snacks, and drinks except water (in the form of photos and text). Physical activity and sleep will also be recorded via a mobile app for step count and sleep time records. The study will be performed at Wenzhou Medical University. The protocol followed the Standard Protocol Items: Recommendations for Interventional Trials statement (27).

### Participant recruitment and screening

Pre-diabetic females and males aged 18–60 years were screened for eligibility according to the inclusion and exclusion criteria, which are listed below in Table 1. Prediabetes was defined as impaired fasting glucose (IFG) and/or impaired glucose tolerance (IGT) and/or an HbA1c of 5.7–6.4%. Subjects were recruited through bulletin board posters and social media advertisements. We sent detailed information by e-mail to anyone interested in this study by e-mail. Prescreening was conducted by questionnaire through personal telephone interviews, in which we recorded age, sex, ethnicity, dietary habits, biological rhythms, sleep, physical activity, family history, etc. If he or she decided to participate in the study, the volunteer signed the written informed consent form, and they were invited to undergo a face-to-face screening at Visit 0 (V0). At V0, subjects underwent a health screening and an oral glucose tolerance test (OGTT). Subjects were informed that their participation is voluntary and that they may withdraw their consent at any time.

**Table 1:** Eligibility criteria.

**Inclusion criteria**
• Aged from 18 to 60 years • FBG 6.1–6.9 mmol/L (IFG) OR 2 h PG (75 g OGTT) 7.8–11.0 mmol/L (IGT) OR HbA1c 5.7–6.4% • Stable bodyweight (changes <5 kg) in the past 3 months • Consumes 3 meals (breakfast, lunch, and dinner) a day at regular time points • Similar levels of physical activity (light manual worker)
**Exclusion criteria**
• Metabolic diseases, including but not limited to diabetes mellitus, cardiovascular disease, kidney or liver disease, hypertension, or hyperuricemia • Special diet requirements, such as vegetarian, or Muslim diets • Treatment with medication that significantly affects glucose metabolism, appetite, or energy balance in the past 3 months • Pregnancy or breastfeeding • Acute or chronic food allergies • Diagnosed or self-reported gastrointestinal disease or discomfort • Shift work or night shift work • Continuous or accumulated smoking for more than 6 months • Daily alcohol drinking
**Withdrawal criteria**
• Participant's withdrawal of informed consent • Noncompliance with consuming the study foods

*FBG, fasting blood glucose; IFG, impaired fasting glucose; PG, postprandial glucose; IGT, impaired glucose tolerance*.

### Randomization and allocation concealment

Stratified randomization will be used. Participants will be stratified by sex. Random number sequences will be generated using SPSS version 24.0 (SPSS Inc. Chicago, IL, USA). A researcher who is independent of the assessment of study outcomes and statistical analysis will perform the random assignments, and grouping code results will be placed in opaque envelopes to ensure group concealment. After inclusion is confirmed, the envelopes will be distributed in the order of inclusion, and the meaning of each group code will not be available to check the grouping status until the beginning of the study. Subjects and staff involved in diet management cannot be blinded, as the participants will consume easily identifiable snacks. Staff assessing clinical outcomes and conducting analysis will remain blinded to the allocation until the statistical analysis is completed.

### Intervention

This study consists of a baseline blood glucose assessment day (D2) under habitual diet conditions (before the start of the intervention, V1), two intervention periods under two different diet patterns with equal experimental days (D3-D6, D10-D13), and a standard balanced meal run-in period between the two intervention periods (D7-D9). A 3-day experiment run-in period will be set before Visit 1 (V1). During this period, all subjects will be asked to maintain their habitual eating without energy restrictions and maintain a regular daily schedule. In addition, they will not be allowed to smoke, drink, or perform vigorous exercise in the course of this trial. All subjects will wear continuous glucose monitoring (CGM) to monitor dynamic interstitial glucose in the mornings of V1. The first intervention period will be under the habitual diet condition from D3 to D6 (4 days), during which all subjects will be asked to maintain their habitual eating and daily activities, similar to the experimental run-in period. After the first intervention period, a 3-day standard balanced meal run-in period from D7 to D9 will be performed before the second intervention to ensure that the standard meals are tolerable to the individual participants. The second intervention will consist of prelunch snacks with standard meals that had the same macronutrient composition as those in the run-in period. Screening will be finished within 1 week before the experiment (V0). Randomization will be performed after the completion of screening. Outcomes will be assessed after the two intervention periods. Blood samples will be taken before and after the two intervention periods (V1-V4), and interstitial glucose will be monitored throughout the 2 week experimental period. An overview of the study visits is presented in Table 2. During the entire period of this experiment, subjects will be asked to complete a food recording diary (online) including the type, amount, and time of all meals, snacks, and drinks except water (in the form of photos and text). Subjects will be supervised daily by the researcher in charge to ensure their food diaries are uploaded on time. Physical activity and sleep will also be recorded via a mobile app for step count and sleep time records.

**Table 2:** Overview of study visits.

**Visit**	**V0**	**V1**	**V2**	**V3**	**V4**
Time from the start of wearing CGM^a^	−7	1	7	10	14
**Participant information**					
Inclusion and exclusion criteria	X				
Informed consent	X				
Medical history (individual and family)	X				
**Outcomes**					
CGM^b^		X	X	X	X
Fasting blood samples		X	X	X	X
HbA1c	X				
VAS^c^	X				
Bodyweight		X	X	X	X
Waist and hip circumference	X	X	X	X	X
Food records^d^	X	X	X	X	X
Physical activity and sleep records	X				
**Questionnaires**					
Sociodemographic characteristics	X				
Eating habits	X				
Acceptability and compliance					X

a *CGM between 8:00 and 9:00 at Visit 1*;

b *collect CGM data from Visit 1 to Visit 4 for 14 consecutive days*;

c *at time points of 10 and 20 min after consumption of the snacks*;

d *records during the entire study*.

*CGM, continuous glucose monitoring; VAS: visual analog scale*.

### Test snacks

In the 3 premeal snack arms, all participants will be given prelunch snacks 30 min before lunch. Prelunch snacks will comprise soy powder and milk powder which will be completely dissolved in 250 ml of water, unsalted roast whole almonds snacks with 250 ml of water, while an equal amount of water (250 ml) will be given to subjects in the control group. The total calories of the three snacks will be similar to each other, from 80 to 90 kcal. This calorie selection was based on the minimum calories needed to relieve the symptoms of hypoglycemia (28). The specific composition of the three food items is shown in Table 3.

**Table 3:** Macronutrient composition of the test snacks.

**Per serving**	**Soy powder**	**Milk powder**	**Almonds**
Weight(g)	18	25	13
Energy (kcal)	83	90	85
Carbohydrate (g, % of energy)	6.2 (30%)	14.0 (62%)	2.7 (12%)
Protein (g, % of energy)	7.2 (35%)	7.5 (33%)	2.7 (12%)
Fat (g, % of energy)	3.0 (32%)	0.38 (3%)	6.8 (72%)

*As an example for soy powder, in this trial, we used a total weight of 18 grams of soy powder as a pre-meal snack, which provided 83 kcal of total energy; of the 18 grams of soy flour, 6.2 g of carbohydrates provided 30% of the total energy, 7.2 g of protein provided 35% of the total energy, and 3.0 grams of lipids provided 32% of the total energy, respectively*.

### Standard balanced meals run-in period

A 3day run-in period will be scheduled between Visit 2 (V2) and Visit 3 (V3), during which subjects will be asked to maintain the same levels of rest and physical activity as they did during the experimental period. Subjects will be given standard balanced meals 3 times per day during the run-in period and will not be provided with premeal snacks. Furthermore, researchers will train the subjects to eat in the same fixed meal order and have a similar duration of each meal (to keep a similar chewing pace) to control for confounding factors.

Standard balanced nutrients will be given for each meal during the second intervention period. Calories will be matched between groups for each meal and a total 24-h period within participants. Energy requirements for the day will be divided by sex calculated with the participants' average using the Harris-Benedict formula (29).

Males: resting metabolic rate (kcal/d) = 66.5 + (13.75 × weight in kilograms) + (5.003 × height in centimeters) − (6.775 × age in years);

Females: resting metabolic rate (kcal/d) = 665.1 + (9.563 × weight) + (1.850 × height) − (4.676 × age).

The energy allocation will be 30, 40, and 30% for breakfast, lunch, and dinner, respectively, with macronutrient ratios following the dietary guideline recommendations of 50–55% of calories from carbohydrates, 15–20% from protein, and 25–30% from fat. Daily meals will include vegetables, meat, and staple foods. After this trial, we will upload the realistic standard meals in the form of a photograph. The types of food are shown in Table 4.

**Table 4:** Food types of standard meals.

**Food type**	**Staple food**	**Vegetable**	**Protein**
Breakfast	Noodles	Tomato	Egg
	Bread	Cucumber	Milk
	Rice	Cabbage	Pork
Lunch and dinner	Rice	Broccoli	Fish
		Cabbage	Chicken
		Carrot	Pork
		Mushroom	Egg
		Tomato	

### Outcomes

#### Primary outcomes

The change in the MAGE before and after the intervention was chosen as the primary outcome for several reasons. First, MAGE is highly correlated with 24-h urinary 8-isoprostane F2α levels, an indicator of oxidative stress in the body, and increased levels of oxidative stress are in turn significantly and positively correlated with HbA1c in patients with type 2 diabetes (30). Second, MAGE is increased with the progression of atherosclerosis in patients with type 2 diabetes combined with atherosclerosis compared to that in those without atherosclerosis (5). The MAGE data calculated by the CGM assay are more accurate and of some reference value. Other indicators of GV, such as the TIR, area under the curve (AUC), variable coefficient (CV), and mean of daily differences (MODD), are also included in the primary outcomes.

#### Secondary outcomes

Secondary outcomes consist of various metabolic and behavioral indicators, including changes in insulin, C-peptide, fasting blood glucose (FBG), HOMA-IR, HOMA-β, subjective appetite, satiety, hunger evaluated by the VAS, body height, and weight measurements.

We will assess changes in these outcomes in two different experimental periods: [1] from baseline to the end of the first intervention period (D3-D6) and [2] from the end of the run-in period to the end of the second intervention period (D10-D13). The acceptability and maintainability of the intervention will also be assessed after the completion of the interventions (D14).

### Outcome measures

#### Test days

All clinical examinations will be carried out at Wenzhou Medical University. Participants will arrive at The 2nd Affiliated Hospital and Yuying Children's Hospital of WMU at 8:00 am after 12 h of overnight fasting (V1). All subjects will be asked to eat the same dinner between 17:00 and 19:00 the day before the experiment to minimize the potential effect of different dinners on fasting blood glucose and insulin levels of the next day (31). In addition, no alcohol consumption or strenuous physical activity will be allowed during the 72 h before the experiment.

#### Continuous glucose monitoring

A 2-week CGM system (FreeStyle Libre Flash Glucose Monitoring System, Abbott) will be initiated on the test day at V1. The CGM will be attached to the hypogastrium on the test day morning. Subjects will be asked to measure their blood glucose levels using a glucometer (glucometer 550, Yuwell) four times a day (before breakfast, lunch, dinner, and bedtime) during the CGM measurement period to calibrate the instruments. The CGM will monitor dynamic blood glucose within the next 15 days. To exclude the potential impact of confounders, the data of dynamic blood glucose from D1, D3, D10, and D14 will be excluded in the final analysis, while data from D2 and D9 will be used as baseline values of two intervention periods. We will only use the data from the latter 3 days of each period rigorously and conservatively to maximally control the potential data contamination.

#### Anthropometry

Body height and weight measurements will be taken using a weight and height scale (JT-918C, J-SKY company, China), under conditions when the subject is wearing only light clothing/underwear. Waist circumference will be measured at the midpoint between the lowest point of the lowest rib and the highest point of the ilium. Hip circumference will be measured at the greater trochanter of the femur. The hip and waist circumferences were averaged by repeating the measurements twice. In the event of a difference of ?3 cm between the two measurements, a third measurement will be taken and the average of the two closest measurements will be used. Body composition (fat mass and skin mass) will be measured using a body composition analyzer (S-ONE, JingHai Health, China).

#### Blood samples

Fasting venous blood samples will be collected via an anterior elbow vein catheter on the four test days (V1-V4) before and after the two intervention periods. Blood samples will be immediately centrifuged for 15 min (3,000 g at 4°C), frozen at −20°C, and analyzed within 30 days of collection. The analysis will include assessment of fasting glucose, fasting insulin, HbA1c, and C-peptide levels. Plasma glucose will be measured by the glucose oxidase technique. Insulin and C-peptide measurements will be performed by electrochemiluminescence.

#### Subjective appetite

The VAS will be used to investigate subjective satiety, hunger, and desire for the next meal (32). After the introduction of the snacks, subjective appetite will be assessed using the VAS at time points of 10 and 20 min. Participants will rate each of the following 4 questions by marking vertically on a 10 cm horizontal line with descriptive anchors on either side (“not at all” to “extremely”): [1] How full do you feel? [2] How hungry do you feel? [3] How strong is your desire to have the next meal? and [4] How much do you think you could (or would you want to) eat right now?

#### Interviews

Face-to-face interviews will be conducted at V4 to evaluate the participants' experiences and perceptions of the intervention in depth. All subjects will be invited to a personal semistructured interview of ~20 min to explore the feasibility and acceptability of the premeal snack intervention being included in their daily dietary habits. It will help to understand the feasibility of the intervention and the acceptability and sustainability of integrating this intervention into their daily lives. If subjects withdraw from this trial, they will be invited to record their reasons for withdrawing and their perceptions of the interventions by a face-to-face interview.

### Comparisons

First, the three intervention arms will be combined as a group and compared with the control group to evaluate whether the premeal intervention could improve GV. Second, the two arms with high-protein snacks will be combined as a group and compared with the control group; moreover, the high-fat snack arm will be compared with the control group to evaluate whether the two snacks (high-protein and high-fat snacks) could improve GV. If both the high-protein and high-fat snacks show significant improvement effects, we will further directly compare the high-protein snack (combining milk powder and soy powder into one group) with the high-fat snack (almonds) interventions to evaluate which is better. Third, we will also compare the milk powder group with the control group and the soy powder group with the control group, and if both high-protein snack groups show significant improvement, we will further compare the animal protein and plant protein groups directly to verify whether the plant protein group is similar to the animal protein group. Finally, we will compare all the results from different dietary patterns to determine whether premeal snacks have similar effects under habitual diet and standard balanced meals.

### Statistical methods

#### Sample size determination

The mean amplitude of glycemic excursions (MAGE) was chosen as the primary outcome indicator and it is expected that the MAGE will be reduced by 20% based on a study by Imai (33). Using PASS software (version 11.0), a sample size of 6 participants per group is required using the non-inferiority test for comparison of sample means. Assuming a 20% dropout rate, the sample size required for this study is 8 participants for each group.

#### Statistical analysis plan

All parameters will be assessed for normality using the Kolmogorov-Smirnov test, and homogeneity of variance will be tested using the Levene test. Descriptive statistics will be expressed as the mean ± standard deviation (SD) for normally distributed data and as the median (Q1-Q3) for non-normally distributed data. Mean changes in parameters within the group will be assessed by calculating the difference between pre and postintervention measurements. When the data are normal and the variance is equal, paired *t*-tests will be used to assess the effect of interventions. Differences between groups will be assessed using the variance ANOVA test. Dunnett's test will be used to determine whether there is a significant difference between the experimental and control groups. When the assumptions for parametric tests are not met in the data, non-parametric statistical methods will be used.

Subgroup analyses by sex will be performed to explore whether the effect is modified by sex. The correlations between physiological factors and blood glucose levels will be assessed by Pearson's or Spearman's correlation analysis. Some *post-hoc* exploratory analyses will also be conducted. All analyses will be performed using SPSS version 24.0 (SPSS Inc. Chicago, IL, USA). The criterion for statistical significance will be a two-tailed *p* < 0.05.

#### Patient and public involvement statement

During the entire experimental period, conversations will be held with the subjects about their experiences, with the aim of understanding and improving their experiences in current and future premeal intervention trials.

## Discussion

This is the first trial that directly assess the effects of three types of natural snacks on GV. One of the primary aims of this study is to determine which type of snacks is better for GV improvement. Short-term and long-term studies showed that high-protein and high-fat snack interventions could improve glucose metabolism, β cell function, lipid profile, satiety, and body weight, etc. (34–38), while previous experiments also reported that high-protein snacks were better at reducing hunger, bringing satiety, and reducing energy intake for the next meal (39). We hypothesize that all three types of snacks could improve GV and perhaps high-protein snacks have a better effect. In addition, most previous studies focused on separate animal-based protein and suggested that premeal animal protein has a superior effect on glycemic control, probably because of the high branched-chain amino acid content of whey protein, which allows for faster digestion and absorption of milk, causing a significant postprandial amino acid response and rapid first-phase insulin release (40, 41). However, none of these studies assessed the effect of plant-based and animal-based high-protein natural snacks on GV. In this trial, we will directly compare the effects of these two types of high-protein snacks and expect to observe a non-inferiority effect of soy powder compared to milk powder. We also hypothesize a non-inferiority effect of plant-based high-protein snacks (whole soy powder) compared with animal-based high-protein snacks (milk powder) on GV improvement. If this hypothesis is proven, the effective use of soy powder could also provide more options for the lactose-intolerant population.

Another important advantage of the trial is that we will implement a premeal snack intervention under two different dietary conditions. Several studies have reported the beneficial effects of premeal snack interventions on glucose metabolism under well-controlled dietary patterns (14, 40, 42). Although the effects observed in this way are significant, such studies under well-controlled conditions could not represent the full extent of this benefit in real daily diet because GV itself can vary with the order of meals, macronutrient ratios, and the interval between meals (11). The purpose of this design is to determine whether the intervention, when applied to a daily diet, will result in improvements similar to those obtained under the experimental control diet. First, before the start of the experiment, we will set a run-in period with the aim of testing the baseline GV levels of the subjects as their own control. After the run-in period, the first period of the intervention will begin, and premeal snacks will be given under exactly the same habitual diet conditions as those in the run-in period, during which no restrictions will be imposed on the amount and type of food consumed by the subjects at their regular meals. After the end of the first intervention period, a standard balanced meal run-in period will be set to ensure that the subjects' metabolism adapts to this meal pattern before the start of the second intervention period, to obtain more stable data for the second-period results.

Notably, as the prediabetic population continues to expand, the solution to reduce the risk of the development of diabetes in this population is an urgent issue. It has been reported that in the process of disorders in glucose metabolism, the increase in postprandial glucose excursion and delayed duration of postprandial glucose excursion occurs earlier than the increase in FBG levels in patients with prediabetes (43). Our study will select subjects from the pre-diabetic population aim to alleviate glucose metabolism and achieve prevention through simple and easily implemented lifestyle interventions. In addition, the results from the prediabetic population are more likely to be extrapolated to diabetic populations.

Furthermore, we will control for confounding factors as strictly as possible. This protocol is based on past studies of the effects of different intervention time points, intervention foods, intervention energies, and intervention populations. However, this study is extended by physical activity level monitoring and daily nutritional recordings (electronic food diary, CGM, and activity records) through timely supervision of the subjects by the researchers and hunger, satiety, and blood glucose and insulin resistance indices will be measured before and after energy intake. During both the ad libitum and standard meal periods, subjects will be asked to record whether and how much food is left over from each meal to assess the effect of the intervention on energy intake at regular meals and to exclude changes in GV due to differences in energy intake. To exclude the influences of different dietary patterns, researchers will train the subjects to eat in the same fixed meal order and have the same duration of each meal (chewing pace) to control for confounding factors in the 3-day balanced meal run-in period. At the end of the experiment, subjects will also be surveyed about their perceptions of the intervention to obtain information about its acceptability, compliance, and adherence. Compared to previous studies, these tools and measures will help avoid confounding factors, advance the understanding of the effect of the premeal snacking intervention on GV and promote the application of the intervention to a wide range of the population. Moreover, we will also administer 250 ml of water to the control group, similar to the 3 intervention groups, to avoid the influences of stomach capacity and swallowing behavior changes on the results (44, 45). In addition, for data processing, we will exclude data from the first day of each phase and the last day of wearing the CGM to exclude data differences caused by the instability of CGM devices.

In conclusion, this is the first RCT protocol that directly compares the effects of different natural snacks under different dietary patterns on GV. We will carry out a careful and rigorous research protocol for the following three issues we want to study: [1] the comparison of GV improvement effects between high-protein snacks and high-fat snacks; [2] the comparison of GV improvement effects between animal-based high-protein snacks and plant-based high-protein snacks; and [3] the comparison of the effects under two different dietary patterns. The limitation of this study is that we will use nuts eaten with water instead of nut powder solution. Because the high-fat powder solution does not have good compliance, we will consider the acceptability of the subjects. Therefore, there may be confounding factors caused by chewing activities in the whole nut group. In addition, the number of people included in this study will be quite small, so the conclusions should be extrapolated with more people. Another limitation of this study is the short duration of the experiment, with only 4 days per period. This is because the maximum validity of a single CGM device is only 14 days. Therefore, the effects we derive will represent only acute effects, and more long-term anthropometric and metabolic outcomes will need to be validated with longer-term experiments.

## Ethics and dissemination

The study was approved by the Medical Ethics Committee of The 2nd Affiliated Hospital and Yuying Children's Hospital of WMU and will be conducted following the Declaration of Helsinki. All equipment used in the study will meet the requirements for subject safety. The total amount of blood taken at each visit will be a maximum of 5 ml and is considered to be safe. The data and biorepository have been approved by the Chinese Data Protection Authority. All research-related information will be recorded, processed, and stored securely in a manner that allows accurate reporting, interpretation, and validation. For CGM measurements, source data will be registered in the web-based software using the subject's study ID. The sponsor/investigator will have direct access to the source data/files for regulatory checks. Anyone can send a request email to the principal investigator to access the full experimental data. Information on injuries/side effects will not be systematically recorded, as we do not expect to cause harm associated with the intervention. All of the positive, negative, or inconclusive findings will be presented at a conference and published in international peer-reviewed journals following the CONSORT guidelines, with no publication restrictions imposed (46).

## Author contributions

YuL, SW, HJ, BR, and XF designed the study. YiL, HJ, and SW prepared the first draft of the manuscript along with the tables and graphs. All authors commented on the manuscript and contributed to the final text. All authors contributed to the article and approved the submitted version.

## Funding

This work was supported by a Wenzhou Medical University grant [89219029].

## Conflict of interest

The authors declare that the research was conducted in the absence of any commercial or financial relationships that could be construed as a potential conflict of interest.

## Publisher's note

All claims expressed in this article are solely those of the authors and do not necessarily represent those of their affiliated organizations, or those of the publisher, the editors and the reviewers. Any product that may be evaluated in this article, or claim that may be made by its manufacturer, is not guaranteed or endorsed by the publisher.
